# Efficacy of Sofosbuvir and Velpatasvir Combination in the Treatment of Hepatitis C Virus (HCV) in Chronic Kidney Disease (CKD) Patients

**DOI:** 10.7759/cureus.60861

**Published:** 2024-05-22

**Authors:** Noor Mohammad, Dilaram Khan

**Affiliations:** 1 Nephrology, Lady Reading Hospital Peshawar, Peshawar, PAK; 2 Gastroenterology, Lady Reading Hospital Peshawar, Peshawar, PAK

**Keywords:** hcv rna, hepatoma, post liver transplant, chronic liver disease, hbv/hcv co-infection, velpatasvir, sofosbuvir, svr12, chronic kidney disease, hepatitis c virus

## Abstract

Background

Hepatitis C virus (HCV) infection is still common in patients with chronic renal failure, even those on maintenance dialysis. A bidirectional association exists between HCV infection and chronic renal disease.

Objective

To assess the efficacy of sofosbuvir and velpatasvir combination in the treatment of chronic HCV in chronic kidney disease (CKD) patients.

Methodology

This descriptive, cross-sectional study was undertaken at the departments of Gastroenterology and Nephrology Lady Reading Hospital, Peshawar, from April 7, 2021, to October 7, 2021. Patients with chronic HCV and chronic renal disease at stage 4 or 5 were included while patients with decompensated cirrhosis liver, hepatoma, hepatitis B virus/HCV (HBV/HCV) coinfection, and post liver transplant patients were excluded. HCV infection was diagnosed based on detectable HCV ribonucleic acid (HCV RNA) by PCR (polymerase chain reaction). In contrast, CKD was diagnosed based on the Kidney Disease Improving Global Outcomes (KDIGO) criteria for CKD. Sofosbuvir 400 mg orally daily and velpatasvir 100 mg orally with meals were given daily for 12 weeks. Effectiveness was defined as negative HCV RNA by PCR 12 weeks after treatment completion called sustained virological response rate 12 weeks after treatment completion (SVR12).

Results

A total of 73 patients including 67 (91.78%) males and six (8.22%) females between the ages of 20 years and 70 years were included in this study. The mean age of the participants was 48.77±8.0 years. Twelve weeks after the treatment completion, 69 (94.52%) had negative HCV RNA, whereas four (5.48%) patients had detectable HCV RNA.

Conclusion

It can be concluded from our study that a fixed-dose combination of sofosbuvir 400 mg and velpatasvir 100 mg is quite effective and recommended for treating chronic hepatitis C infection in patients with chronic renal disease in our local setup.

## Introduction

The global prevalence of hepatitis C virus (HCV) infection has increased to more than 180 million persons (about 2.8% of the global population) [1]. HCV is transmitted from one person to another mainly through percutaneous routes such as blood transfusions, syringe reuse, surgery, hospitalization, piercing, and barber shaving. Pakistan lacks a countrywide hepatitis surveillance system, highlighting the significance of HCV as a public health issue in the country [2]. Pakistan is placed second after Egypt on the list of nations with the greatest prevalence of HCV infection of 4.5-8.2% [3].

HCV infection is linked to the development of chronic kidney disease (CKD) [4], and in people with chronic HCV, there is also an elevated risk of progression to end-stage renal disease (ESRD) [5]. The presence of renal insufficiency is defined as an estimated glomerular filtration rate (eGFR) of less than 45 mL/min/1.73 m2 (chronic renal disease stage 3b, 4, or 5) [6]. HCV has many genotypes, with genotype 3a (GT3a) being the most frequent (69.1%) in Pakistan, followed by GT1 (genotype-1) (7.1%), GT-2 (4.2%), and 4 (2.2%) [7]. Regardless of HCV genotype, patient demographics, or other disease attributes, the fixed-dose combination of sofosbuvir and velpatasvir, an NS5A inhibitor, provides an intervention option for HCV-infected patients [8].

Although the sofosbuvir/velpatasvir combination has been licensed for use in Pakistan since March 2018, it is not covered under the National Hepatitis-Control Program. There is no national directly acting antiviral (DAA) drug distribution program. Therefore, pharmaceutical companies confront strong generic competition [9]. Velpatasvir is an inhibitor of the HCV NS5A protein with antiviral activity against all HCV genotypes that have recently been explored in conjunction with sofosbuvir.

A once-daily, fixed-dose combination pill (sofosbuvir/velpatasvir) achieved exceptionally excellent rates of sustained virological response (SVR) when given for 12 weeks among previously treated and untreated people infected with HCV genotypes 1-6, including those with induced or degenerative cirrhosis, for 12 weeks [10]. The Food and Drug Administration (FDA) has licensed these medications called direct-acting antivirals, which can achieve more than 90% SVR rates in patients infected with HCV [11].

Sofosbuvir (GS-7977), a nucleotide analog NS5B polymerase inhibitor, is a prodrug that is metabolized in the liver to its pharmacologically active form, uridine analog triphosphate GS-461203, which can incorporate into the HCV ribonucleic acid (HCV RNA) by NS5B polymerase resulting in termination of RNA synthesis ceasing viral replication. Dephosphorylation results in the formation of the nucleoside inactive metabolite GS-331007; although inactive, it is the primary product analyzed for pharmacokinetic data. Following oral administration, peak plasma concentration is seen within 0.5 to two hours post dose for sofosbuvir and within two to four hours for GS-331007. Following a single 400 mg oral dose, 80% is eliminated in the urine, and 14% is eliminated in the feces. GS-331007 is excreted by the kidneys and accumulates five- to 20-fold in people with stage 4-5 CKD (including those on dialysis) [12]. Because of the above findings, the current study was conducted at the departments of Gastroenterology and Nephrology, Lady Reading Hospital, Peshawar, to investigate the efficacy and safety of sofosbuvir and velpatasvir combination in the treatment of hepatitis C in CKD to treat those CKD patients who are having HCV to prevent cirrhosis and decompensation of another vital organ of the body.

## Materials and methods

Study design

This descriptive study comprising 73 patients was conducted at the departments of Gastroenterology and Nephrology at Lady Reading Hospital, Peshawar, from April 7, 2021, to October 7, 2021, using a nonprobability consecutive sampling technique, and the sample size was calculated taking the prevalence of HCV as 4.5-8%, confidence level as 95%, and the margin of error as 5%[3].

Patients of either sex with chronic hepatitis C child class A and CKDs in stage 4 (GFR of 15-30 mL/min) and stage 5 (GFR of less than 15 mL/min) between the ages of 20 and 70 years were included in the study, while patients with chronic liver disease Child-Pugh classes B and C, hepatoma, concomitant hepatitis B infection, and post liver transplant patients were excluded from the study.

Operational definitions

Child class A cirrhosis was defined as patients who were well compensated (i.e., having a Child-Pugh score of 5-6). Child class B cirrhosis was defined as patients who had significant functional compromise (i.e., having a Child-Pugh score of 7-9), while child class C was defined as those patients who had decompensated disease by having a Child-Pugh score of 10-15.

Data collection procedure

Patients were enrolled in the institute’s indoor gastroenterology and nephrology sections baseline demographics of the patients were noted. Patients' clinical history and clinical examinations were done and all necessary investigations were carried out. CKD was confirmed using the Kidney Disease Improving Global Outcomes (KDIGO) criteria comprised of patients having urine albumin to creatinine ratio (ACR) more than 30 mg/gm, eGFR less than 15 mL/min/1.73 m^2^, and renal biopsy revealed glomerulosclerosis in more than 50% of the glomeruli, interstitial fibrosis, tubular atrophy, interstitial mononuclear infiltrates, arteriolar hyalinosis, and arterial intimal thickening in more than 50% of the region.

Hepatitis C was diagnosed by doing HCV RNA by polymerase chain reaction from the hospital laboratory and was taken positive or detectable when more than 50 IU/mL were present. Besides the HCV RNA and renal function tests, other laboratory tests such as complete blood counts, liver function tests, liver synthetic functions, and ultrasound abdomen were done before starting the patients on antiviral drugs.

All patients included in the study received fixed-dose combination tablets containing 400 mg sofosbuvir and 100 mg velpatasvir orally with meals daily for 12 weeks. The patients were followed up initially on a weekly basis for one month and then bi-weekly with physical exams and necessary blood tests such as complete blood count and liver function tests. Quantitative HCV RNA was done 12 weeks after treatment completion (also called SVR12) by polymerase chain reaction (PCR) from the hospital laboratory. Those who had less than 50 IU/mL of HCV RNA or undetectable HCV RNA as determined by PCR were considered to have achieved SVR12/treatment responders, while those who had more than 50 IU/mL of HCV RNA were labeled as nonresponders.

Data analysis

We used Statistical Product and Service Solutions (SPSS, version 22; IBM SPSS Statistics for Windows, Armonk, NY) to analyze the data. Categorical variables were assessed as frequencies (percentages), whereas continuous variables were recorded as medians with ranges. The level of significance was considered as a P value of ≤ 0.05.

Ethical consideration

Proper approval was taken from the ethical review board of the hospital-wide letter No. 101/Lady Reading Hospital/Medical Teaching Institute (dated 07/04/2021) and ensured that it complied with all applicable ethical standards. Patient information anonymity was ensured, and all study procedures followed the guidelines outlined in the Declaration of Helsinki.

## Results

The study comprised 73 patients, including 67 (91.78%) males and six (8.22%) females. The minimum age was 20 years, while the maximum age was 70 years in this study, with a mean age of 48.77±8.0 years. The majority of the patients were in the age range of 41-50 years (Table 1).

**Table 1:** Demographics and disease characteristics of patients (n=73) HCV: hepatitis C virus

Variables	Frequency	Percentage (%)
Age		
20-30	2	2.74
31-40	15	20.55
41-50	27	36.99
51-60	15	20.55
61-70	14	19.18
Total	73	100.00
Mean Value: X̅ = 48.77; Standard deviation ± 8.0		
Gender		
Male	67	91.78
Female	6	8.22
Total	73	100.00
Kidney disease stage		
4	60	82.19
5	13	17.81
Total	73	100.00
Duration of hemodialysis		
<5 years	65	89.04
>5 years	8	10.96
Total	73	100.00
HCV duration		
<1 year	64	87.67
>1 year	9	12.33
Total	73	100.00

Stage 4 kidney disease was found in 60 (82.19%) patients, while stage 5 disease was present in 13 (17.81%) patients. In 65 (89.04%) patients, the duration of hemodialysis was less than five years, while eight (10.96%) patients were having hemodialysis for more than five years. In 64 (87.67%) patients, HCV was present for less than one year, while in nine (12.33%) patients, it was present for more than one year as shown in Table 1.

Multiple laboratory tests were performed before starting a combination of sofosbuvir/velpatasvir treatment to assess kidney function and HCV replicative and liver status (Table 2).

**Table 2:** Mean values of laboratory tests before and after 12 weeks of using a fixed dose of sofosbuvir/velpatasvir (n=73) ALT: alanine transaminase; AST: aspartate transaminase; eGFR: estimated glomerular filtration rate; HCV RNA: hepatitis C virus ribonucleic acid

Variables	Before treatment, mean±SD	After treatment, mean±SD
Hemoglobin (g/dL)	9.14±0.51	13.5±1.75
Total leukocyte count (/mm^3^)	65,000±2,045	75,000±2,000
Direct bilirubin (mg/dL)	0.8±0.1	0.6±0.1
ALT (U/L)	57.34±49.52	44.4±1.9
AST (U/L)	32.01±34.01	43.7±1.2
Serum protein	6.16±0.18	7.0±1.0
Serum albumin (mg/dL)	3.68±0.21	5.1±1.1
Platelet count (lac/mm^3^)	222.6±56.54	183.5±74.5
Total bilirubin (mg/dL)	1.9±0.21	1.03±1.45
eGFR (mL/min/1.73 m^2^)	43.1±1.21	60.1±9.5
HCV RNA IU/mL	13.25±1.2	21.25±2.1
Serum creatinine (mg/dL)	1.9±0.2	1.12±0.2

Sixty-nine (94.52%) patients had negative HCV RNA at 12 weeks (SVR12) after treatment completion (treatment responders), whereas four (5.48%) patients had detectable HCV RNA 12 weeks after treatment completion (nonresponders) (Table 3).

**Table 3:** Efficacy of sofosbuvir/velpatasvir in the treatment of HCV in CKD patients (n=73)

Particulars	Frequency (f)	Percentage (%)
Sustained Virological Response 12		
Achieved	69	94.52
Not achieved	4	5.48
Total	73	100.00

A chi-square test was performed to check the association between the efficacy of sofosbuvir/velpatasvir and patient demographics/disease characteristics and the result showed that the patients on hemodialysis for a longer period had a lower chance of recovering. Similarly, the length of hepatitis C duration had a direct association with the efficacy of DAA as a P value<0.05 as shown in Table 4.

**Table 4:** Association of demographic data and efficacy of sofosbuvir/velpatasvir among CKD patients (n=73)

Variables	Sustained virological response at 12 weeks posttreatment		X^2^ P value
	Achieved	Not achieved	
Age			0.39
20-30	2	0	
31-40	15	0	
41-50	25	2	
51-60	15	0	
61-70	12	2	
Gender			0.94
Male	63	4	
Female	6	0	
Kidney disease stage			0.39
4	58	2	
5	11	2	
Duration of hemodialysis			
<5 years	65	0	0.00
>5 years	4	4	
HCV duration			
<1 year	64	0	0.00
>1 year	5	4	

## Discussion

ESRD patients with HCV infection are a particular population that needs antiviral therapy for the treatment of HCV infection. Sofosbuvir and velpatasvir are pan-genotypic regimens for the treatment of HCV infection [13]. The data on the efficacy of this regimen show that ESRD is scant in our local setup. This study was done to ascertain the efficacy of the sofosbuvir and velpatasvir combination in patients with chronic hepatitis C (CHC) and ESRD. Desnoyer et al. investigated the plasma levels of sofosbuvir and its metabolite in hemodialysis patients and concluded that they did not build up during the treatment or in between hemodialysis sessions [14].

In this descriptive study analyzing the effectiveness of sofosbuvir and velpatasvir combination for the treatment of HCV in CKD patients, we observed an SVR12 achievement rate in 69 (94.52%) patients, which is almost comparable to the study done by De et al. where an SVR was achieved in about 97% patients [13]. Similarly, the results of our study are also nearly comparable to the study done by Mandhwani et al. where they used sofosbuvir-based antiviral drugs for the treatment of HCV in end-stage renal disease patients and achieved an SVR12 in 97% of patients [15].

Our study results are also comparable to the study results done by Gaur et al. in India wherein an SVR12 was achieved in 96.8% of participants of the study [16] and to the results of a study done by Shahid et al. in Sheikh Zayed Hospital Rahim Yar Khan, wherein SVR was achieved in 91% CKD patients who were having an HCV infection and were treated with sofosbuvir and velpatasvir combinations [17]. The findings of this descriptive study are also consistent with the study results in efficacy, done by Borgia et al. where they enrolled 59 CKD patients having chronic hepatitis C infection, and a combination of sofosbuvir and velpatasvir was given, and 56 (95%) patients achieved SVR12 [18].

From a systematic study, interferon-based medicines had low efficacy/safety in CKD, while the introduction of direct-acting antiviral medications has resulted in a paradigm shift in the treatment of HCV infection. Elbasvir/grazoprevir, glecaprevir/pibrentasvir, and sofosbuvir-based regimens are now recommended for the treatment of HCV in advanced CKD (including patients on continuous dialysis). There is no need to modify the dosage, and the treatment lasts 8-12 weeks. However, current statistics show that many patients with advanced CKD are still untreated, and there are several impediments to HCV antiviral treatment [19].

Limitations

The main limitations of our study are descriptive study design, small sample size, and its status as a single-center study. However, our study will provide local evidence on this important topic.

## Conclusions

Hepatitis C infection is prevalent in patients with CKD, especially in those on hemodialysis. Our study has shown that a combination of sofosbuvir and velpatasvir is quite effective in patients with CKD. Approximately 95.4% of patients achieved SVR in our study, which is comparable to the efficacy of directly acting antivirals in patients without CKD.
